# Insights into the Mechanism of Action of Phosphoantigen Prodrugs on Vγ9Vδ2 T Cell Activation

**DOI:** 10.1021/acsptsci.6c00264

**Published:** 2026-09-01

**Authors:** Maria Sharif, Claudia M. A. Pinna, Kamila Rakhimova, Taher E. Taher, Andrew D. Beggs, Youcef Mehellou, Benjamin E. Willcox

**Affiliations:** † Cancer Immunology and Immunotherapy Centre, College of Medicine and Health, 1724 University of Birmingham, Edgbaston, Birmingham B15 2TT, U.K.; ‡ School of Infection, Inflammation and Immunology, Department of Immunology and Immunotherapy, College of Medicine and Health, University of Birmingham, Edgbaston, Birmingham B15 2TT, U.K.; § School of Medical Sciences, Department of Cancer and Genomic Sciences, College of Medicine and Health, University of Birmingham, Edgbaston, Birmingham B15 2TT, U.K.; ∥ University Hospitals Birmingham NHS Foundation Trust, Birmingham B15 2GW, U.K.; ⊥ School of Pharmacy and Pharmaceutical Sciences, Cardiff University, Cardiff CF10 3NB, U.K.; # Medicines Discovery Institute, Cardiff University, Cardiff CF10 3AT, U.K.; ∇ National Institute for Health and Care Research (NIHR), Birmingham Biomedical Research Centre, Birmingham B15 2TH, U.K.

**Keywords:** Vγ9Vδ2 T cells, prodrug, ProPAgen, mechanism, uptake, cancer

## Abstract

The activation of Vγ9Vδ2 T cells has emerged as a promising modality in cancer immunotherapy. Phosphate and phosphonate prodrugs of Vγ9Vδ2 T cell phosphoantigens have been developed as potent activators of Vγ9Vδ2 T cells that are capable of effectively facilitating lysis of a range of cancer cells *in vitro*. In this work, we show that phosphonate prodrugs of the phosphoantigen derivative, HMBP, enter cells in an energy-independent manner and produce their pharmacological activity independently of the mevalonate pathway, a target of the widely used bisphosphonate and Vγ9Vδ2 T cell activator, zoledronate. Interestingly, these prodrugs, termed HMBP ProPAgens, induced an enhanced and more universal Vγ9Vδ2 T cell cytotoxicity against diverse cancer cell lines *in vitro* compared to zoledronate, an effect also observed in patient-derived colorectal cancer organoids. Together, the data presented in this work further support the development of HMBP ProPAgens as a novel class of cancer immunotherapeutic agents.

## Graphical abstract

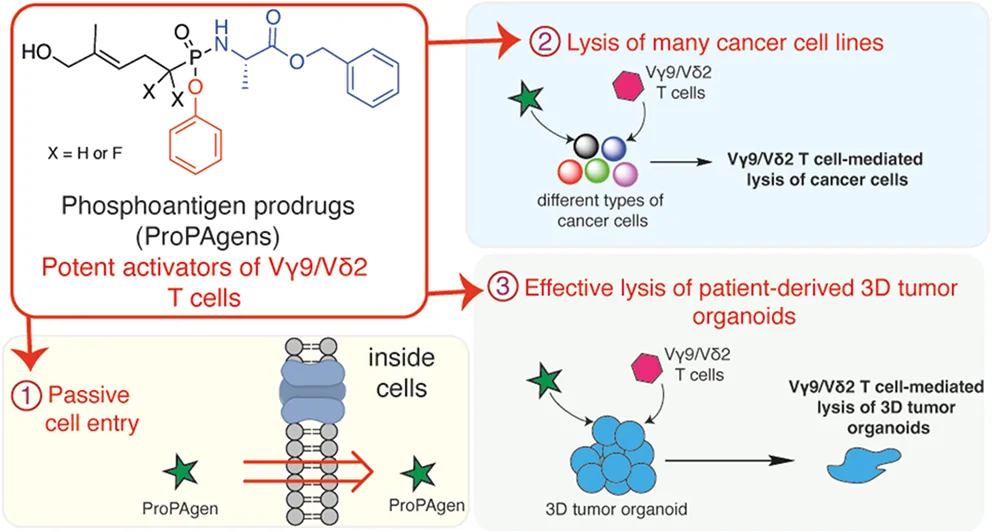

As a predominant subset of human γδ T cells, Vγ9Vδ2 T cells have the ability to recognize and kill a diverse range of cancer cells *in vitro* and *in vivo* models.^1–3^ Vγ9Vδ2 T cells are activated by small-molecule phosphoantigens (pAgs) such as the potent microbially derived antigen (*E*)-4-hydroxy-3- methyl-but-2-enyl pyrophosphate^4^ (HMBPP) and the considerably less potent, host-derived molecule isopentenyl pyrophosphate^5^ (IPP) (Figure 1a). These pAgs activate Vγ9Vδ2 T cells by binding to the intracellular B30.2 domain of the type-1 transmembrane protein butyrophilin 3A1 (BTN3A1) on target cells,^6,7^ which acts as a key component of the “pAg sensor.” pAg binding to BTN3A1 critically alters the cytosolic domain of BTN3A1 ^7,9^ and triggers its association^8,9^ with that of BTN2A1,^10,11^ which ultimately coordinates their ectodomains as a composite ligand^12,13^ capable of binding to and triggering the Vγ9Vδ2 T cell receptor (TCR), in a Major Histocompatibility Complex (MHC)-independent-manner.^5,14^ TCR triggering typically results in potent effector responses by Vγ9Vδ2 T cells, including cytotoxicity and production of IFN-γ and TNFα.^15,16^

**Figure 1:**
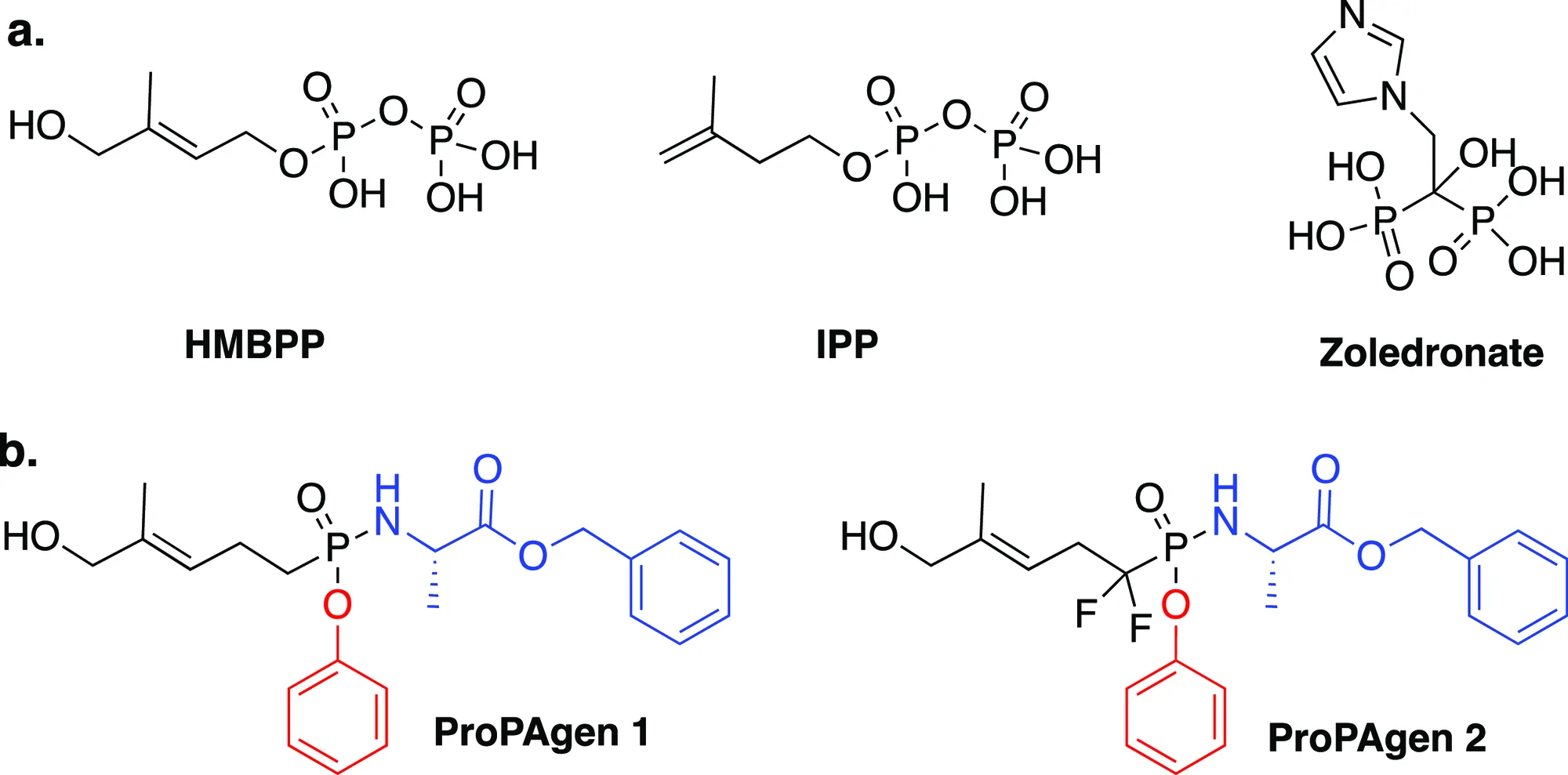
Chemical structures of Vγ9Vδ2 T cell activators. (a) Structures of Vγ9Vδ2 T cell pAgs HMBPP and IPP; also zoledronate, which activates Vγ9Vδ2 T cells by inhibiting IPP catabolism. (b) Chemical structures of our two HMBP ProPAgens. These agents exhibit varying Vγ9Vδ2 T cells activation potency; HMBPP (EC_50_ = 60–500 pM),^17–19^ IPP (EC_50_ = 1–10 μM),^19,20^ zoledronate (EC_50_ = 0.003–0.5 μM),^18,21^ ProPAgen **1** (EC_50_ = 4.7 fM),^21^ and ProPAgen **2** (EC_50_ = 9.15 fM).^21^

Given Vγ9Vδ2 T cells’ universal presence in humans, their proliferative capacity, potential implication in tumor-specific immunity, MHC-unrestricted target cell recognition mode, and pharmacological manipulability with small molecules, they have become a major focus for the development of γδ T cell therapeutics.^2,3^ To date, the pharmacological manipulation of Vγ9Vδ2 T cells has largely been achieved either using their pAgs such as HMBPP or agents that modulate the mevalonate pathway and inhibit the catabolism of the host-derived pAg IPP, such as zoledronate (Figure 1a). However, these compounds are associated with poor drug-like properties due to the presence of highly charged pyrophosphate or diphosphonate groups, which limit cellular uptake and favorable distribution, and which can be susceptible to dephosphorylation. To overcome this, we^18,21,22^ and others^17,23,24^ have developed prodrug-modified forms of these pAgs, which we have termed ProPAgens, as a means of improving their drug-like properties. In these prodrugs, the monophosphonate derivative of the HMBPP ligand was used, and the negative charges of the monophosphonate group were covered by (1) *bis*-pivaloyloxymethyl (*bis*POM),^17^ (2) aryloxy diester phosphoramidates,^21,23^ (3) aryl and pivaloyloxymethyl,^24^ or diamidate^22,25^ groups. These efforts led to the generation of ProPAgens that exhibited excellent serum stability, potent activation of Vγ9Vδ2 T cells, and effective lysis of cancer cells *in vitro* via direct Vγ9Vδ2 T cell-mediated cytotoxicity.^21,23^ Examples of these are the aryloxy diester phosphoramidate prodrugs of HMBP, exemplified by ProPAgens **1** and **2** (Figure 1b), in which the phosphonate group was masked by a phenyl group and l-alanine benzyl ester motifs that are cleaved intracellularly to release the pAg HMBP phosphonate.^21^ These ProPAgens induced superpotent activation of Vγ9Vδ2 T cells, demonstrating EC_50_ values of 4.7 fM and 9.15 fM, respectively, and this resulted in effective lysis of the human urinary bladder cancer cell line T24.^21^

Although phosphoramidate prodrugs, especially those of nucleoside monophosphates, have been shown to enter cells via passive diffusion due to their small size and lipophilic nature, the cellular uptake pathway of these HMBP ProPAgens has not been experimentally established. Additionally, evidence establishing these ProPAgens are able to activate Vγ9Vδ2 T cells independently of the mevalonate pathway akin to zoledronate’s mechanism of action is currently lacking. Moreover, questions regarding both the selectivity of these ProPAgens for target cells and their ability to trigger lysis of solid tumor cells in 3D culture by Vγ9Vδ2 T cells remain unresolved. Aiming to address these outstanding questions, we herein show that HMBP ProPAgens are taken into cells via passive diffusion and exhibit their pharmacological activity independently of the mevalonate pathway. Additionally, we show that HMBP ProPAgens trigger a Vγ9Vδ2 T cell cytotoxic response that promotes lysis of diverse cancer cell lines. Critically, we show that this HMBP ProPAgen-induced Vγ9Vδ2 T cell cytotoxicity can result in lysis of patient-derived 3D organoid tumors.

## Results and Discussion

### HMBP ProPAgens Enter Cells via Passive Diffusion and Activate Vγ9Vδ2 T Cells Independent of the Mevalonate Pathway

Although HMBP ProPAgens (Figure 1b) have been reported to activate Vγ9Vδ2 T cells,^18,21^ experimental evidence regarding their mechanism of uptake into cells and how their activity relates to the mevalonate pathway, a target for the extensively studied Vγ9Vδ2 T cell activator zoledronate, is currently lacking. To address this, we initially sought to understand whether HMBP ProPAgens enter cells via passive diffusion akin to how aryloxy ester prodrugs of nucleoside monophosphates and monophosphonates enter cells.^26,27^ For this, the ovarian cancer cell line SKOV3 was pretreated with 10 μM zoledronate, 10 μM HMBPP, or 10 nM of ProPAgens **1** and **2** at either 4 or 37 °C for 2 h. The choice of the lower temperature, 4 °C in this case, was driven by the fact that the incubation of cells at low temperatures inhibits energy-dependent uptake mechanisms.^28^ The cells from each treatment were then cocultured with zoledronate-expanded Vγ9Vδ2 T cells from healthy donors to assess cytotoxicity in a short-term europium-based assay (Figure 2a). Vγ9Vδ2 T cell cytotoxicity from the 10 μM zoledronate-treated SKOV3 decreased from 25.4% at 37 °C to 9.7% at 4 °C. For the pAg HMBPP, there was a higher level of tumor sensitization at both temperatures when compared to zoledronate, although this decreased significantly from 62.4% at 37 °C to 30.8% at 4 °C (*p* = 0.015). Collectively, these data indicate that zoledronate and HMBPP are likely to enter cells via active uptake, namely fluid-phase endocytosis or pinocytosis, which can be reduced or inhibited by low temperatures.^28^ These observations are consistent with findings from previous studies where the tumor sensitization capacities of zoledronate and HMBPP were reduced by incubation at low temperature (e.g., 4 °C).^29,30^

**Figure 2:**
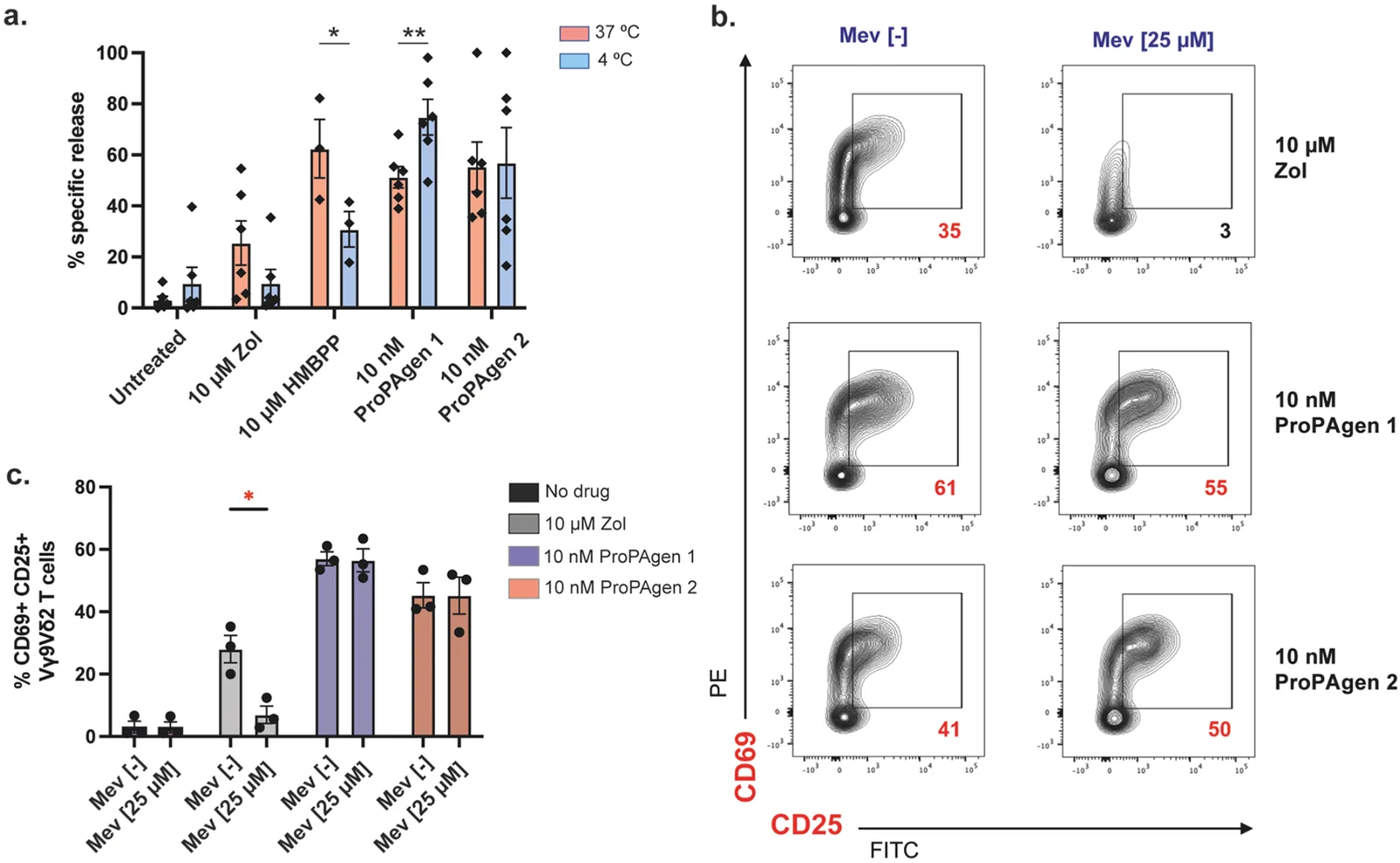
Mechanism of action of HMBP ProPAgens. (a) Cytotoxicity of zoledronate-expanded Vγ9Vδ2 T cells toward SKOV3 ovarian tumor cells treated with zoledronate, HMBPP, and ProPAgens **1** and **2** at 4 and 37 °C. SKOV3 ovarian cells were treated with the drugs at 4 °C (blue bars) and 37 °C (pink bars) for 2 h, and cytotoxicity from ZOL-expanded Vγ9Vδ2 T cells was assessed using a Europium assay. Data for % specific release (i.e., level of killing of SKOV3 cells by *ex vivo* expanded Vγ9Vδ2 T cells) are shown as mean ± SEM of *n* = 6 donors for untreated, zoledronate, ProPAgens **1** or **2**, and *n* = 3 for HMBPP PAg. Statistical analysis was carried out via GraphPad Prism v9 with a two-way ANOVA and Šidák’s multiple comparisons test. (b) Activation assays were performed overnight in the presence and absence of 25 μM Mevastatin (Mev), as well as 10 μM zoledronate, 10 nM ProPAgen **1** or ProPAgen **2**. Representative flow cytometry plots showing coexpression of activation markers CD69 and CD25 in one donor. (c) Mean %CD69+ CD25+ Vγ9Vδ2 T cells ± SEM (*n* = 3). Data were analyzed via FlowJo v10 and GraphPad Prism v9. Statistical analysis was carried out via a two-way ANOVA with Šidák’s multiple comparisons test. *p = 0.0162.

For the HMBP ProPAgens, however, decreased temperature had no negative impact on Vγ9Vδ2 T cell cytotoxicity, based on comparison of 10 nM HMBP ProPAgens **1** and **2** treatments at 4 and 37 °C. Indeed, the Vγ9Vδ2 T cell cytotoxicity at 4 °C with ProPAgen **2**-treated SKOV3 cells was maintained at ∼55%, but ProPAgen **1**, on the contrary, showed an increase in killing of SKOV3 cells when used at 4 °C compared to 37 °C (increase from 51.3% at 37 °C to 74.8% at 4 °C; *p* = 0.01). The lack of decrease in Vγ9Vδ2 T cell cytotoxicity observed with the HMBP ProPAgens **1** and **2** treatments at 4 or 37 °C suggests that these ProPAgens’ uptake into cells is energy-independent. It is notable that this conclusion was drawn when ProPAgens **1** and **2** were used at 10 nM concentrations (EC_50s_ = 0.00000497 nM and 0.00000915 nM, respectively), 10 μM HMBPP (EC_50_ = 0.145 nM) and 10 μM zoledronate (EC_50_ = 3.06 μM).^21^ Nevertheless, our observation herein that ProPAgens **1** and **2** enter cells via passive diffusion is in line with previous studies where *bis*-pivaloyloxymethyl prodrugs of HMBP were also found to produce their pharmacological effects independently of temperature changes from 4 to 37 °C, indicating their passive cellular uptake.^29^

Notably, with our HMBP ProPAgens, there was a statistically significant increase in the levels of killing of SKOV3 cells pretreated with ProPAgen **1** at 4 °C in comparison to the same coculture where SKOV3 cells were treated at 37 °C. It is unclear why ProPAgen **1** was able to sensitize ovarian SKOV3 cells more effectively at 4 °C than 37 °C as the activity of the enzymes that metabolize these prodrugs might be negatively impacted by the colder temperatures. However, this notion was challenged previously where enzymatic activity as low as 30% was found to still lead to sufficient metabolic activity of this type of prodrug, likely due to the great abundance of the metabolic enzymes inside cells.^31^ Nevertheless, our data collectively confirm that zoledronate and HMBPP enter cells via active uptake, in agreement with previous reports,^29^ and establish that in contrast, our HMBP ProPAgens are able to enter cells via passive diffusion and sensitize the host cells toward Vγ9Vδ2 T cell cytotoxicity.

Next, we asked whether the Vγ9Vδ2 T cell cytotoxicity induced by our HMBP ProPAgens could also involve the mevalonate pathway, similar to the mechanism of action of zoledronate. To answer this, we compared the Vγ9Vδ2 T cell cytotoxicity induced by zoledronate or our HMBP ProPAgens **1** and **2** in the presence and absence of mevastatin (Figure 2b,c). Mevastatin is a competitive inhibitor of 3-hydroxy-3-methylglutaryl coenzyme A (HMG-CoA) reductase, an enzyme in the early stages of the mevalonate pathway,^16,32^ and thus prevents biosynthesis of IPP, which activates Vγ9Vδ2 T cells, thereby rendering zoledronate ineffective. With this in mind, healthy donor peripheral blood mononuclear cells (PBMCs) were incubated either untreated (in medium only), or treated with 10 μM zoledronate, or with 10 nM of ProPAgens **1** or **2**, in both the presence and absence of 25 μM mevastatin. Subsequently, Vγ9Vδ2 T cell activation was assessed by CD69 and CD25 expression, both of which are upregulated upon TCR stimulation as previously reported.^15^ As expected, treatment with 25 μM mevastatin abrogated the ability of zoledronate to activate Vγ9Vδ2 T cells (Figure 2b). The average proportion of CD69+ CD25+ Vγ9Vδ2 T cells decreased from 28% in zoledronate-only treated samples to 7% in the presence of 25 μM mevastatin when the cells were stimulated with 10 μM zoledronate (*p* = 0.016; Figure 2c). In fact, the levels of Vγ9Vδ2 T cell activation reached a value very close to that of the untreated controls (ca. 3% CD69+ CD25+ in both mevastatin-treated and untreated wells). On the contrary, treatment with 25 μM mevastatin had no impact on 10 nM ProPAgens **1**- and **2**-treated PBMCs, with the proportion of CD69+ CD25+ Vγ9Vδ2 T cells remaining at ca. 57% and ca. 45%, respectively. These results confirm the hypothesis that HMBP ProPAgens are able to activate Vγ9Vδ2 T cells independently of the intracellular mevalonate pathway, whereas zoledronate, as shown previously,^33^ relies heavily on the flux through this pathway in order to exert its effects on Vγ9Vδ2 T cells.

Such data distinguish the mechanism of action of our HMBP ProPAgens from zoledronate, a widely used bisphosphonate and Vγ9Vδ2 T cell activator. This is an important finding as the activity of the mevalonate pathway can vary depending on the type of cancer and the requirement for, for example, cholesterol production to maintain membrane integrity and viability.^34,35^ Previous studies indicated that tumors can have dysregulated mevalonate pathway flux via either deficient feedback control of HMG-CoA reductase enzyme, or increased expression and activity of this enzyme.^36,37^ In addition, dysregulation of this metabolic pathway can lead to cell transformation, thereby suggesting that HMG CoA reductase may be a potential oncogene.^38^ Therefore, dependence of the cancer cell on flux through the mevalonate pathway, in combination with energy-dependent uptake of zoledronate into cells, could be key reasons for the variable activity of zoledronate in sensitizing different tumor cells to Vγ9Vδ2 T cell-mediated killing. As our HMBP ProPAgens enter cells passively and operate independently of the mevalonate pathway, whose activity can vary across cancer cells, it is plausible to suggest that HMBP ProPAgens are likely to trigger more universal Vγ9Vδ2 T cell cytotoxicity across cancer cell lines than zoledronate.

### ProPAgen-Induced Vγ9Vδ2 T Cell Cytotoxicity Across Cancer Cell Lines

To investigate whether HMBP ProPAgens do trigger a more universal Vγ9Vδ2 T cell cytotoxicity than zoledronate, we assessed the ability of zoledronate and HMBP ProPAgens **1** and **2** to sensitize a range of target tumor cells; the human bladder cancer cell lines T24, UMUC3 and 5637, as well as ovarian cancer lines CAOV3 and SKOV3, toward killing by *ex vivo* zoledronate-expanded Vγ9Vδ2 T cells (Figure 3a and Supporting Figure S1). Initially, the target cancer cells were sensitized in the absence of effector Vγ9Vδ2 T cells. No significant background cell death was observed in all target tumor cells treated with 10 μM zoledronate or 10 nM ProPAgens **1** or **2** (% spontaneous release at <10%, Supporting Figure S1). With T24 cells, a slightly elevated background killing with 10 nM ProPAgen **2** (% spontaneous release = 15.7) was observed, but this was not statistically significant. However, when the cells were treated with zoledronate or the HMBP ProPAgens for 2 h followed by addition of *ex vivo* zoledronate-expanded Vγ9Vδ2 T cells, killing of the various cancer cells was observed (Figure 3a). Notably, the levels of cancer cell lysis varied across the cancer cell lines depending on the drug treatment and the individual Vγ9Vδ2 donor.

**Figure 3:**
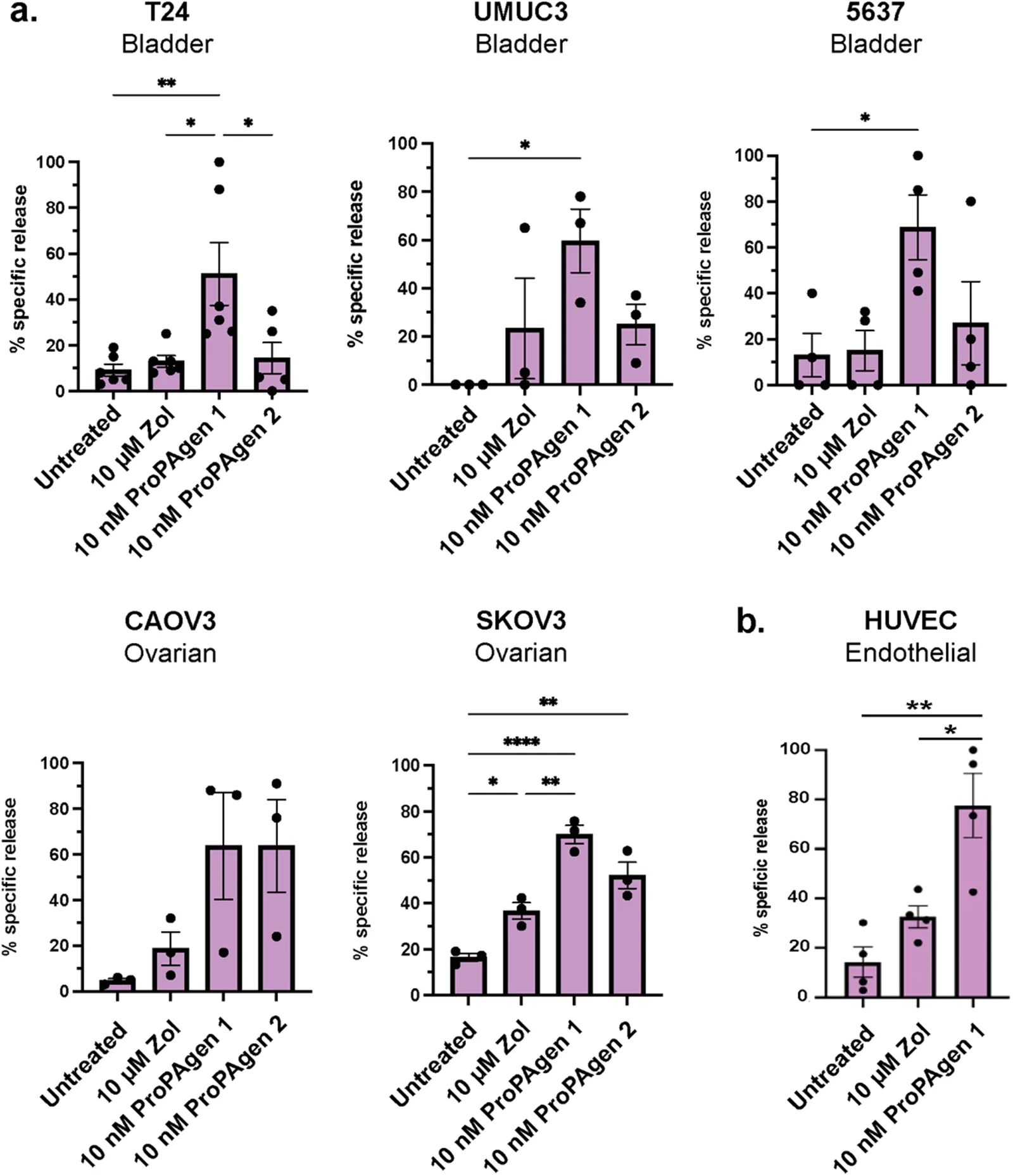
Cytotoxicity of zoledronate-expanded Vγ9Vδ2 T cells toward zoledronate- and ProPAgen-treated tumor cell lines and HUVEC cells. The cell lines were treated with ZOL and ProPAgens for 2 h at 37 °C, and cocultured with Vγ9Vδ2 T cells for 1 h at an effector:target ratio of 80:1. (a) Vγ9Vδ2 T cell-mediated killing of target cells, with data shown as mean ± SEM of *n* = 6 (T24), *n* = 3 (UMUC3, CAOV3 and SKOV3), and *n* = 4 (5637). Statistical analysis was performed using ordinary one-way ANOVA with Tukey’s multiple comparisons test on GraphPad Prism v9 software. *p < 0.05 **p < 0.005 ****p < 0.0001. (b) Europium cytotoxicity assay was carried out with HUVEC healthy cells, where they were either untreated or pretreated with 10 μM zoledronate or 10 nM ProPAgen **1** for 2 h as described earlier. % specific release is a measurement of killing of HUVEC target cells. Data are shown as mean ± SEM (*n* = 4), and all analyses were carried out via GraphPad Prism v9 software, with statistical tests performed with ordinary one-way ANOVA with Tukey’s multiple comparisons test.

Given that zoledronate’s ability to sensitize target cells for Vγ9Vδ2-mediated lysis is dependent upon mevalonate pathway flux as discussed above, it was expected that treating various cancer cells with zoledronate would result in variable Vγ9Vδ2 T cell-mediated killing as the activity of the mevalonate pathway likely varies between the different cancer cell lines studied in this work (Figure 3a).^34,35^ Indeed, as shown in Figure 3a, levels of killing for 10 μM zoledronate-treated target cells resulted in 13.0, 23.3, 15.0, 18.7 and 36.7% for T24, UMUC3, 5637, CAOV3 and SKOV3 cell lines, respectively. In addition, treatment of these cell lines with 10 μM zoledronate did not result in significantly higher levels of killing when compared to the untreated controls (*p* > 0.05), with one exception being SKOV3, where % specific release increased from 16.6% in untreated control to 36.7% in 10 μM zoledronate -treated sample (*p* = 0.03), with very little donor variation relative to the other cell line cocultures. Regarding ProPAgens **1** and **2**, only ProPAgen **1** showed a consistent significant increase in the killing of treated tumor cells when compared to 10 μM zoledronate-treated and untreated controls. The levels of killing of 10 nM ProPAgen **1**-treated tumor targets were in the range of 50–83% for all cell lines, with significant differences observed in comparison to the untreated controls, and in some instances to 10 μM zoledronate- and 10 nM ProPAgen **2**-treated targets. Surprisingly, despite having very similar potency for Vγ9Vδ2 T cell activation *in vitro*, ProPAgen **2** was unable to sensitize all tumor lines akin to that observed with ProPAgen **1**; a killing range lower and much broader (14–80%) in comparison to its nonfluorinated relative ProPAgen **1**. Admittedly, the difference between ProPAgens **1** and **2** in the efficacy of cancer cell killing, especially in T24, UMUC3, and 5637 cancer cells (Figure 3a) is intriguing since both ProPAgens displayed equally potent activation of Vγ9Vδ2 T cells in PBMC assays.^18^ The difference in sensitization of bladder cancer cell lines observed here could be attributable to differential rates of metabolism of the two ProPAgens, affecting release of the active phosphoantigen in these cell lines under the assay conditions used. Notably, in these cytotoxicity assays, the effector:target ratio was 80:1 in line with our previous reports^22^ though it is worth mentioning that ProPAgens **1** and **2** retain significant sensitization of the bladder cancer T24 cell line at effector:target ratios of 1:1 and 8:1,^21^ akin to the data shown in Figure 3a.

Having established universal HMBP ProPAgen **1**-mediated Vγ9Vδ2 T cell cytotoxicity across the various cell lines studied, we then explored whether such cytotoxicity can be observed toward noncancer cells. To probe this, we studied the ability of ProPAgen **1** to sensitize nontransformed human umbilical vascular endothelial cells (HUVECs), as target cells, to Vγ9Vδ2 T cell-mediated cytotoxicity in cocultures with zoledronate or ProPAgen **1**. For this, HUVECs were used either untreated or pretreated with 10 μM zoledronate or 10 nM ProPAgen **1** in the same manner as in the cocultures with tumor cell targets. Although average killing in the presence of zoledronate was increased compared to untreated target cells, as in previous coculture assays, there were no significant differences between the cytotoxic capacity of zoledronate-treated and untreated HUVEC cells. (Figure 3b). However, pretreatment of HUVEC cells with 10 nM ProPAgen **1** led to a ∼5.4-fold increase in Vγ9Vδ2 T cell-mediated killing compared to the untreated controls, and a 2.7-fold increase compared to zoledronate-treated targets, respectively.

The data suggested that both drugs can, to an extent, sensitize noncancer cells for Vγ9Vδ2 T cell-driven cytotoxicity, with ProPAgen **1** leading to a substantially greater sensitization of the healthy targets than zoledronate. It must also be noted that this set of data was obtained in *in vitro* assays, which might be different to how these compounds act *in vivo*. Indeed, previous clinical studies in humans in which an HMBPP analogue was used *in vivo* to activate Vγ9Vδ2 cells indicated such activation was safe in humans.^39,40^ This safety profile suggests that *in vivo*, activated Vγ9Vδ2 T cells may have an increased level of selectivity toward tumors than noncancer cells.

### Lysis of Patient-Derived Colorectal Cancer 3D Organoids by HMBP ProPAgen-Induced Vγ9Vδ2 T Cell Cytotoxicity

After establishing the wide-spectrum Vγ9Vδ2 T cell cytotoxicity that is triggered by HMBP ProPAgens in cell culture, we wanted to study such HMBP ProPAgen-induced Vγ9Vδ2 T cell cytotoxicity in 3D organoid models as these are arguably more physiologically relevant than 2D cell culture. Additionally, the use of 3D organoid models for studying Vγ9Vδ2 T cell antitumor effects could provide some hints regarding the ability of these lymphocytes to infiltrate 3D solid tumors, which 2D cell culture models do not provide.

For our 3D organoid studies, we chose to conduct these experiments in a single patient-derived primary colorectal cancer (CRC) organoid. The choice of CRC as a focus for the 3D organoid studies is driven by the need for new treatments for this disease, which is the third most prevalent cancer and was responsible for >880,000 deaths worldwide in 2018,^41^ and the fact that Vγ9Vδ2 T cell activation has been shown to have favorable CRC antitumor activity.^42^

Akin to our above studies in cancer cell lines, CRC organoids were either seeded untreated or were pretreated with 10 μM zoledronate or 10 nM ProPAgen **1** for 2 h, and then cocultured overnight in the presence of CFSE-labeled Vγ9Vδ2 T cells and Annexin V RED dye (Figure 4a). The levels of green and red fluorescence were then assessed with an EVOS fluorescence microscope (Figure 4b), with the former reflecting CFSE-labeled Vγ9Vδ2 T cells, and the latter the amount of red fluorescence deriving from dying organoid cells, which was quantified via ImageJ (Figure 4c).

**Figure 4:**
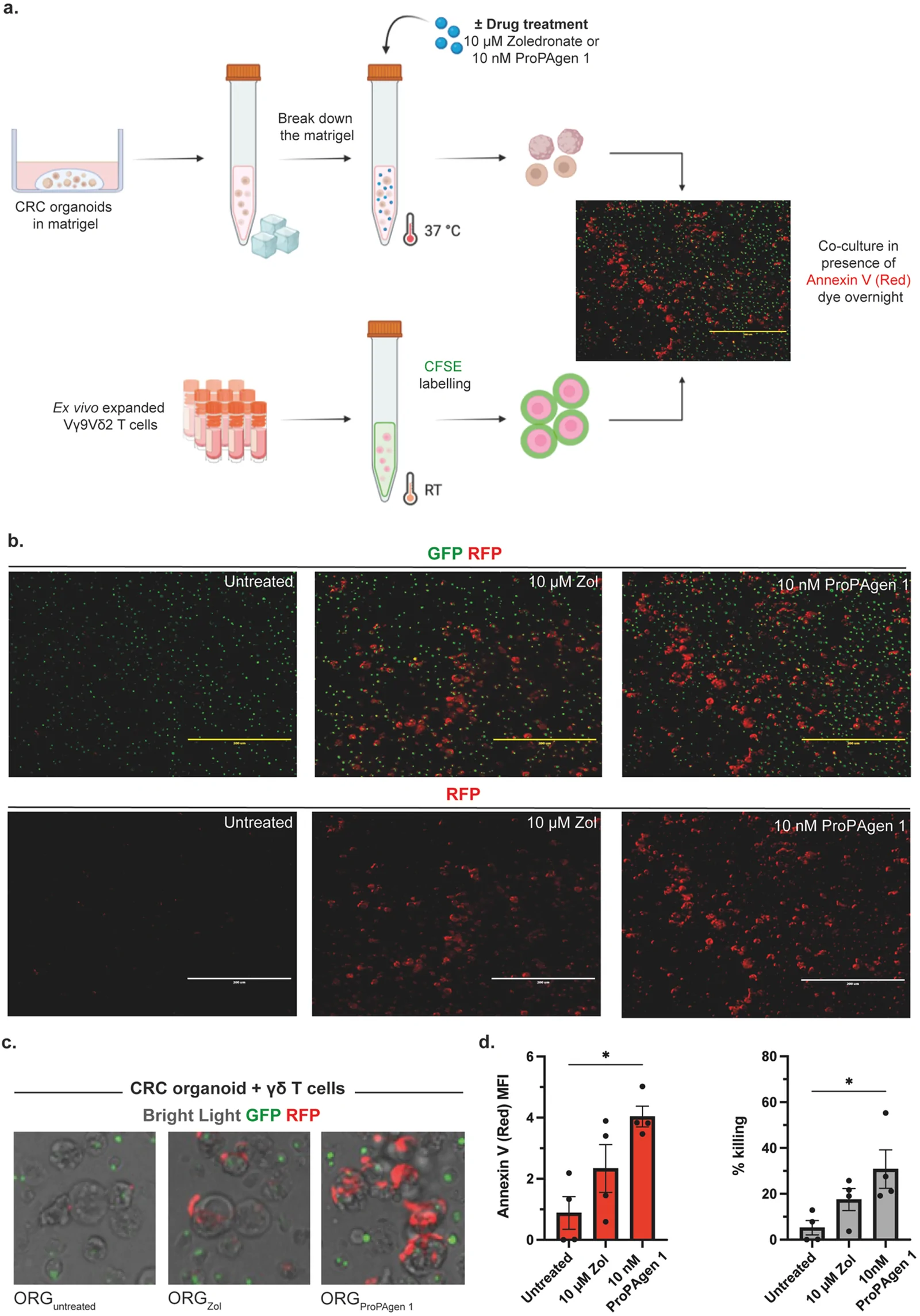
HMBP ProPAgen 1 sensitizes CRC organoids better than zoledronate for Vγ9Vδ2 T cell-mediated killing. *Ex vivo* expanded Vγ9Vδ2 T cells were labeled with CFSE and cocultured with independent CRC organoids that were either untreated or pretreated with 10 μM Zol or 10 nM ProPAgen **1** for 2 h. The coculture was performed overnight in the presence of Annexin V RED dye. The cocultures were then imaged via an EVOS imaging microscope in bright light, GFP and RFP channels. All fluorescence was quantified with ImageJ software and plotted as the MFI of Annexin V RED. (a) Schematic illustration of the coculture protocol. (b, c) Images of the cocultures with GFP and RFP channels combined, and RFP alone; magnification = X20; scale bar = 200 μm. (d) Red fluorescence plotted as MFI, and % killing of the organoids in the cocultures calculated as follows: 100­[(coculture MFI–organoids alone MFI)/(DMSO+ organoid MFI–organoids alone MFI)]; organoids cultured in the presence of DMSO were used as a positive control for lysis with the highest red fluorescence MFI. Data were analyzed with ImageJ and GraphPad Prism v9 software. Statistical analysis was carried out via one-way ANOVA with Tukey’s multiple comparisons test; *p* = 0.01 (MFI) and 0.03 (% killing).

As expected, and in line with our previous data from the cell line cytotoxicity assays, the level of organoid killing increased consistently from untreated to 10 μM zoledronate- and 10 nM ProPAgen **1**-treated CRC samples, and was approximately 2-fold greater in cocultures with ProPAgen **1** pretreated samples than those pretreated with zoledronate (Figure 4b–d). Overall, the organoid coculture data demonstrated very similar patterns of cell killing to data obtained from the cytotoxicity assays obtained with tumor cell lines, indicating that the prodrug pretreatment strategy can be applicable in a patient-proximal tumor setting.

In conclusion, HMBP ProPAgens have been shown to be potent activators of Vγ9Vδ2 T cells, and their activation resulted in significant cytotoxicity toward a small selection of cancer cells *in vitro*. Herein, we provided key insights into the cellular uptake and mechanism of action of these ProPAgens, which distinguished them from the bisphosphonate class of Vγ9Vδ2 T cell activators, as exemplified by zoledronate. Indeed, we found that, in contrast to zoledronate, HMBP ProPAgens enter cells in an energy-independent manner, and upon their metabolism, they activated Vγ9Vδ2 T cells independently of the mevalonate pathway. Notably, we also observed that the Vγ9Vδ2 T cell-mediated cytotoxicity toward different cancer cells caused by HMBP ProPAgens was more powerful and universal than that induced by zoledronate. Such potent Vγ9Vδ2 T cell-mediated cytotoxicity was also noted in colorectal cancer organoids, suggesting that HMBP ProPAgens may have utility in treating solid tumors. Given their broad mode of action, exploring their potential use in cancers of unmet need such as pancreatic cancer would be attractive, particularly as pancreatic cancer cells have been shown to be relatively resistant to γδ T cell attack, which can be overcome to an extent by phosphoantigens.^43^ This warrants further investigation. Nevertheless, increased sensitization of untransformed HUVEC cells suggests increased intracellular bioavailability comes with a greater potential for on-target off-tumor toxicity, and thus care should be exercised in devising administration strategies, or conceivably drug modification approaches, that take this into account. Collectively, this work strengthens the case for pursuing further investigations into the *in vivo* safety and efficacy of HMBP ProPAgens as promising immunotherapeutics against a variety of cancers.

## Experimental Section

### Cell Lines and Cell Culture

T24, 5637, and CAOV3 cells were maintained in RPMI supplemented with 10% fetal calf serum (FCS) and 1% penicillin-streptomycin (Pen-Strep). UMUC3 cells were cultured in DMEM 10% FCS, 1% Pen-Strep, and SKOV3 cells in McCoy’s 5A 1× (Modified) medium with 10% FCS, 1% Pen-Strep. Human umbilical vascular endothelial cells (HUVEC) were purchased from ATCC and cultured in F12K medium supplemented with 20% FCS, 2 mM l-glutamine, 0.1 mg/mL heparin, and 30 μg/mL ECGS (endothelial cell growth supplement). All cells were passaged regularly once the cell density reached >70% confluence.

### Isolation of Healthy Donor PBMC

All healthy donors participating in this study were aged over 21 and were recruited from the Institute of Immunology and Immunotherapy, University of Birmingham. Written informed consent was obtained from all donors prior to any blood withdrawal, with ethics approval granted by the London Harrow Research Ethics Committee (IRAS ID: 300402; REC reference 21/PR/1068; approved on 24.11.2021). Healthy donors participating in this study were recruited through the University of Birmingham, or healthy donor whole blood was obtained from the NHS Blood Transfusion Center (NHSBT). Peripheral blood (PB) was collected, and PB mononuclear cells (PBMCs) were extracted via a Ficoll gradient method using Lymphoprep reagent (STEMCELL Technologies). After the final wash step, PBMCs were resuspended in RPMI supplemented with 5% human AB serum (HABS), 1% Pen-Strep, and 1% Glutamax medium (T cell medium).

### Expansion of Vγ9Vδ2 T Cells from Healthy Donor PBMC

PBMCs were cultured for a period of 14 days in order to expand Vγ9Vδ2 T cells. Cells were seeded (2 × 10^6^ cells/mL) and expanded in 24-well plates in T cell medium and supplemented with 5 μM zoledronate and 100 U/mL interleukin-2 (IL-2). Cells were then incubated at 37 °C/5% CO_2_ and fed with fresh media containing 100 U/ml IL-2 every 2–3 days until day 14 of culture. Expanded cells were harvested and frozen down at the end of the 14-day culture to be used for cytotoxicity assays. Vγ9Vδ2 T cell purity was assessed by flow cytometry, comparing day 0 and day 14 of culture.

### Flow Cytometry

To assess purity of Vγ9Vδ2 lymphocytes during *ex vivo* expansion, cells were stained at days 0 and 14 of culture with the following panel in MACS buffer (1X PBS, 2% FCS, 5 mM EDTA): Live–Dead (Zombie Aqua; Biolegend), CD3 (BV421; Biolegend), Vγ9 (PEcy5; Beckman Coulter), Vδ2 (APC; Miltenyi Biotec). To assess Vγ9Vδ2 T cells in overnight activation assays, cells were stained with the same panel as that used for examining Vγ9Vδ2 T cell purity during expansion, but with the addition of antibodies for CD69 (PE; Beckman Coulter) and CD25 (FITC; Biolegend) activation markers. After staining, cells were washed in MACS buffer and fixed in IC fixation buffer (Invitrogen). Finally, the fixation buffer was washed off and cells were centrifuged at 600 rcf for 5 min. All cells were resuspended in 200 μL MACS buffer for acquisition on Fortessa (BD Biosciences). All data were obtained by FACSDiva software (BD Biosciences) and analyzed using FlowJo v10.

### Europium-Based Cytotoxicity Assay

To assess cytotoxicity of *ex vivo* expanded Vγ9Vδ2 T cells toward drug-treated and untreated tumor cell lines, a short-term europium-based cytotoxicity assay was carried out using the DELFIA EuTDA Cytotoxicity kit (PerkinElmer). For this, target SKOV3 cells were harvested, counted, and resuspended in PBS to a cell concentration of 1 × 10^6^ cells/mL. Two batches of tubes were set up, one for drug treatment at 4 °C and one for 37 °C. Cells were transferred into 15 mL tubes at 1 mL cells per tube and incubated either at 37 °C/5% CO_2_ or at 4 °C for a period of 2 h in the following conditions for each temperature setting: cells with no drug (untreated control), cells with 10 μM zoledronate, and cells with 10 nM ProPAgen. After the drug treatment, cells were washed in PBS, resuspended in PBS containing 1 μL/mL BATDA loading reagent, and incubated at 37 °C/5% CO_2_ for 20–40 min. Simultaneously, *ex vivo* expanded Vγ9Vδ2 T cells were thawed for the assay. 100 μL of target cells (5000 cells) were seeded per well into a U-bottom 96-well plate and cocultured with 100 μL of Vγ9Vδ2 T cells (400,000 cells) per well in T cell medium to give an effector:arget ratio of 80:1. For target-only controls (spontaneous release with no effectors), 100 μL per well of culture medium was added instead, and for the positive killing (maximal release) control, 10% lysis buffer was added. The plate was centrifuged at 200 rcf for 2 min and incubated at 37 °C/5% CO_2_ for 1 h. Twenty-five μL of supernatant was collected after the incubation and centrifuging the plate again. The supernatants were transferred into optical flat-bottom 96-well plates, and 200 μL Europium solution was added per well. The plate was read on a PHERAstar microplate reader (BMG Labtech) to measure time-resolved fluorescence. The % killing (also referred to as % specific release/lysis) of target tumor cells was calculated by the following formula:

%specificrelease=100×[(experimentalrelease−spontaneousrelease)/(maximalrelease−spontaneousrelease)]

The data were analyzed via Microsoft Excel and GraphPad Prism v9 software. For T24, UMUC3, 5637, CAOV3 and HUVEC cell cytotoxicity assays, the same method was applied, but the cells were treated with zoledronate and ProPAgens for 2 h at 37 °C/5% CO_2_ only.

### Activation of Vγ9Vδ2 T Cells

Healthy donor PBMCs were thawed, counted, and seeded out in T cell medium into U-bottom 96-well plates at 5 × 10^5^ cells in a total volume of 200 μL of T cell medium per well (unstimulated) or stimulated with 10 μM zoledronate or 10 nM ProPAgens, and cultured overnight at 37 °C/5% CO_2_. Twenty-five μM Mevastatin (Sigma) was added to investigate the impact of blocking the intracellular mevalonate pathway on drug-induced activation of Vγ9Vδ2 T cells. Activation was assessed on the following day by flow cytometry.

### Primary CRC Organoid Culture

Patient-derived colorectal cancer (CRC) organoids were provided by Professor Andrew D. Beggs (University of Birmingham). These organoids were obtained from a single patient (diagnosis: stage IV primary transverse colon tumor and ulcerative colitis) and were used due to immediate availability, as well as good sample viability and growth. The organoids were grown in 24-well tissue culture plates in “BME domes” consisting of a 3:1 ratio of basement membrane extract type-2 (BME2; Bio-Techne) to Intesticult culture medium (STEMCELL Technologies). Each well contained 500 μL Intesticult, which was replaced every 2–3 days, and the organoids were passaged weekly when they reached 200–300 μm in diameter. The organoid size was assessed via an EVOS imaging microscope (Invitrogen) at 10× and 20× magnifications. To passage the organoids, media was removed from each well and 500 μL ice-cold PBSO (PBS without Ca^2+^/Mg^2+^; Thermofisher Scientific) was added to each well. The BME domes with the organoids were transferred into 15 mL tubes, centrifuged at 4 °C, and the supernatant was removed. The organoids were dissociated in TrypLE containing 10 μM Rho kinase inhibitor (RhoK; R&D Systems) and incubated in a 37 °C water bath for 5–10 min. Once dissociated, cold PBSO was added to wash the cells. Supernatant was removed, and the organoid cells were resuspended in cold PBSO and counted. Approximately 0.1 × 10^6^ organoid cells were cultured per 50 μL BME dome, and the remaining organoid cells were discarded. The organoids were either grown for γδ T cell coculture experiments or frozen down once they reached ca. 200 μm in diameter.

### Primary CRC Organoid Killing Assay

CRC organoids that reached ca. 100–200 μm in diameter were harvested from their BME domes by removing the culture medium, adding Cell Recovery Solution (Corning), transferring into 15 mL tubes, and incubating on ice for 1 h. Once the BME domes were dissociated, the tubes were centrifuged, and the organoids were resuspended in PBSO. As with the cell lines previously, organoids were either incubated untreated (in medium only) or were pretreated with medium containing 10 μM zoledronate or 10 nM ProPAgen for 2 h at 37 °C/5% CO_2_. During this period, *ex vivo* expanded Vγ9Vδ2 T cells were thawed and labeled with 0.1 μM CFSE (Abcam) for 20 min at room temperature in the dark. The cells were then washed twice in warm T cell medium and resuspended to 2 × 10^6^ cells/mL. CRC organoids were washed in cold PBSO twice and resuspended in T cell medium. 100 μL of organoids were seeded into nontissue culture flat-bottom 96-well plates, and cocultured overnight in the presence of 100 μL CFSE-labeled Vγ9Vδ2 T cells and 1:800 diluted Annexin V RED dye (Sartorius) per well. Organoid-only controls were also seeded with 100 μL culture medium added instead of the T cells plus medium. A positive organoid death control (maximal killing) was also set up with organoids cultured in medium containing 20% DMSO. The level of fluorescence was assessed with an EVOS imaging microscope, with red fluorescence deriving from the dying organoids quantified via ImageJ software. In addition, the level of organoid killing was calculated as the percentage killing using a similar formula as with the Europium assay, and the results were plotted and analyzed with GraphPad Prism v9 software.

## Supplementary Material



## Notes

Drug-induced cytotoxicity against cancer cell lines in the absence of Vγ9Vδ2 T cells (Figure S1) (PDF)

## Abbreviations

BTN2A1: butyrophilin 2A1
BTN3A1: butyrophilin 3A1
HMBP: (*E*)-4-hydroxy-3-methylbut-2-enyl monophosphate
HMBPP: (*E*)-4-hydroxy-3-methylbut-2-enyl pyrophosphate
IPP: isopentenyl pyrophosphate
PAg: phosphoantigen
ProPAgen: prodrug of a phosphoantigen
TCR: T-cell receptor
