# Supplementary material for: Insights into the Mechanism of Action of Phosphoantigen Prodrugs on Vγ9Vδ2 T Cell Activation
Source: ACS Pharmacol Transl Sci. 2026 Sep 1;9(9):2585–94. doi: 10.1021/acsptsci.6c00264 (PMC13632888; doi:10.1021/acsptsci.6c00264)
Supplement: Supplementary file 1 [file pt6c00264_si_001.pdf]

Insights into the Mechanism of Action of Phosphoantigen Prodrugs on Vγ9Vδ2 T Cell Activation

Maria Sharif,[1,2] Claudia M. A. Pinna,[3] Kamila Rakhimova,[3,4] Taher E. Taher,[1,2] Andrew D. Beggs,[3,4] Youcef Mehellou,[5,6*] Benjamin E. Willcox[1,2,7*]

[1]*Cancer Immunology and Immunotherapy Centre, College of Medicine and Health, University of Birmingham, Edgbaston, Birmingham B15 2TT, U.K.*
[2]*School of Infection, Inflammation and Immunology, Department of Immunology and Immunotherapy, College of Medicine and Health, University of Birmingham, Edgbaston, Birmingham B15 2TT, U.K.*
[3]*School of Medical Sciences, Department of Cancer and Genomic Sciences, College of Medicine and Health, University of Birmingham, Edgbaston, Birmingham B15 2TT, U.K.*
[4]*University Hospitals Birmingham NHS Foundation Trust, Birmingham B15 2GW, U.K.*
[5]*School of Pharmacy and Pharmaceutical Sciences, Cardiff University, Cardiff CF10 3NB, U.K.*
[6]*Medicines Discovery Institute, Cardiff University, Cardiff CF10 3AT, U.K.*
[7]*National Institute for Health and Care Research (NIHR), Birmingham Biomedical Research Centre, Birmingham B15 2TH, U.K.*

**Emails:* MehellouY1@cardiff.ac.uk and b.willcox@bham.ac.uk

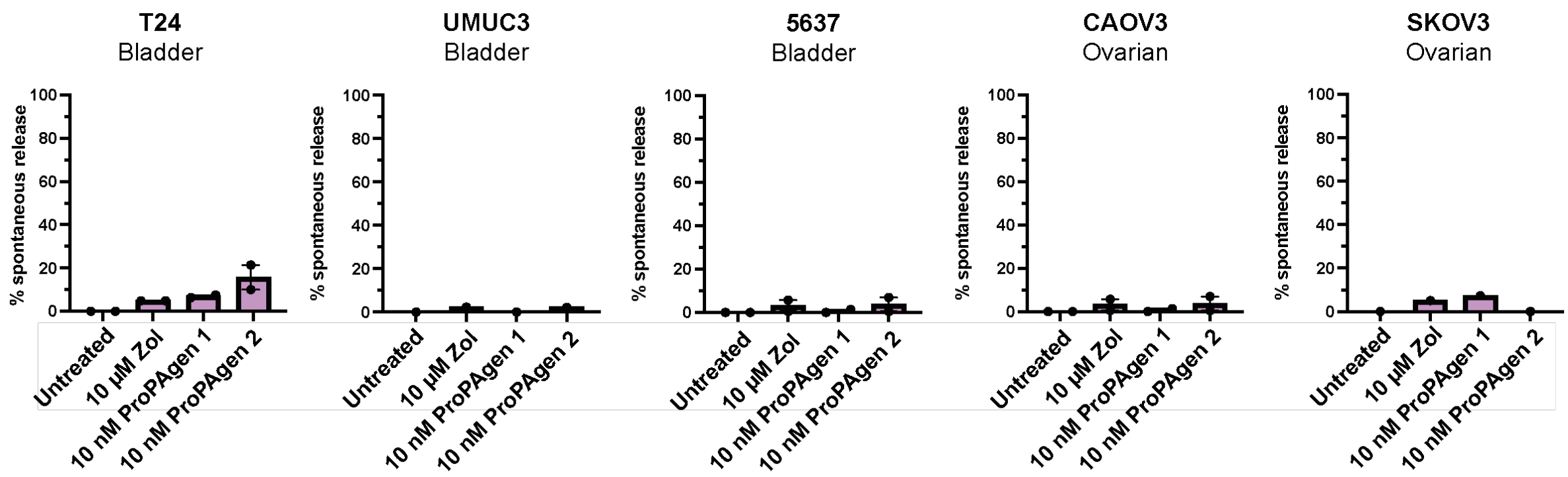

Figure S1. Cytotoxicity of zoledronate and ProPAgens 1 and 2 towards cancer cell lines. The cell lines were treated with zoledronate (Zol) and ProPAgens **1** or **2** for 2 h at 37 °C. Cytotoxicity was determined using the Europium-based cytotoxicity assay. n = 1 for UMUC3 and SKOV3, and n = 2 for T24, 5637 and CAOV3.
